# The Importance of Taking a Patient-Centered, Community-Based Approach to Preventing and Managing Frailty: A Public Health Perspective

**DOI:** 10.3389/fpubh.2020.599170

**Published:** 2020-11-12

**Authors:** Kadjo Yves Cedric Adja, Jacopo Lenzi, Duygu Sezgin, Rónán O'Caoimh, Mara Morini, Gianfranco Damiani, Alessandra Buja, Maria Pia Fantini

**Affiliations:** ^1^Department of Biomedical and Neuromotor Sciences, University of Bologna, Bologna, Italy; ^2^School of Nursing and Midwifery, College of Medicine Nursing & Health Sciences, National University of Ireland Galway, Galway, Ireland; ^3^Mercy University Hospital, Grenville Place, Cork, Ireland; ^4^Clinical Research Facility Cork, University College Cork, Cork, Ireland; ^5^Italian Scientific Society of Hygiene and Preventive Medicine – Primary Care Group, Bologna, Italy; ^6^Fondazione Policlinico Universitario A. Gemelli IRCCS, Rome, Italy; ^7^Università Cattolica del Sacro Cuore, Rome, Italy; ^8^Laboratory of Health Care Services and Health Promotion, Evaluation Unit of Hygiene and Public Health Department of Cardiac Thoracic Vascular Sciences and Public Health, University of Padova, Padua, Italy

**Keywords:** public health, primary care, frailty, biopsychosocial (BPS) model multidisciplinary, holistic care course

## Abstract

Across the world, life expectancy is increasing. However, the years of life gained do not always correspond to healthy life years, potentially leading to an increase in frailty. Given the extent of population aging, the association between frailty and age and the impact of frailty on adverse outcomes for older people, frailty is increasingly being recognized to be a significant public health concern. Early identification of the condition is important to help older adults regain function and to prevent the negative outcomes associated with the syndrome. Despite the importance of diagnosing frailty, there is no definitive evidence or consensus of whether screening should be routinely implemented. A broad range of screening and assessment instruments have been developed taking a biopsychosocial approach, characterizing frailty as a dynamic state resulting from deficits in any of the physical, psychological and social domains, which contribute to health. All these aspects of frailty should be identified and addressed using an integrated and holistic approach to care. To achieve this goal, public health and primary health care (PHC) need to become the fulcrum through which care is offered, not only to older people and those that are frail, but to all individuals, favoring a life-course and patient-centered approach centered around integrated, community-based care. Public health personnel should be trained to address frailty not merely from a clinical perspective, but also in a societal context. Interventions should be delivered in the individuals' environment and within their social networks. Furthermore, public health professionals should contribute to education and training on frailty at a community level, fostering community-based interventions to support older adults and their caregivers to prevent and manage frailty. The purpose of this paper is to offer an overview of the concept of frailty for a public health audience in order to raise awareness of the multidimensional aspects of frailty and on how these should be addressed using an integrated and holistic approach to care.

## Introduction

The world is rapidly aging. According to the United Nations Department of Economic and Social Affairs “*World Population Aging 2019*” report, there are currently 703 million people aged ≥65. This is projected to reach 1.5 billion, representing one in every six people, by 2050, up from one in every 11 in 2019 (1). Similarly, the number of people aged ≥80 years will triple in the next 30 years and life expectancy after 65 years of age is predicted to increase by 19 years (1). However, the years of life gained do not always correspond to healthy life years. The latest figures, published for 2020 by Eurostat, reveal that in the European Union (EU) the proportion of healthy life years represents approximately 76.7 and 81.4% of the total life expectancy for women and for men, respectively (2). The reduction in healthy life years is characterized by an increase in frailty, multimorbidity and disability (3), which results in frequent use of healthcare services by older adults (4). All the above-mentioned conditions can impair several domains of health (i.e., physical, psychological, cognitive, and social), requiring holistic care for complex needs that are the consequence of multiple determinants of health.

The purpose of this paper is to offer an overview of the concept of frailty for a public health audience, in order to provide a better understanding of the condition and its prevention and management. Specifically, this paper aims to raise awareness of the multidimensional aspects of frailty and how these should be addressed using an integrated and holistic approach to care. To achieve this goal, public health and primary health care (PHC) need to become the fulcrum through which care is offered, not only to older people and those that are frail, but to all individuals, favoring a life-course and patient-centered approach focused on integrated community-based care.

## Frailty as a Multidimensional Construct

Frailty is a complex age-associated syndrome without a universally accepted definition (5–7). It results from a decrease in multiple physiological systems that leads to a state of increased vulnerability to stressor events with impaired ability to achieve homeostasis (8–10). Such triggers can include worsening of a chronic condition (11), environmental factors (12), change in therapies (3), and adverse life events (13). In older adults a progressive loss of physiological reserve is characteristic of frailty (14). Frailty is also associated with an increasing risk of adverse outcomes (3, 15–17), which includes falls (18, 19), fractures (20), disability (18, 19), delirium (21), depression (22), cognitive impairment (21), hospitalizations (18, 23, 24), need for long-term care (19), poor quality of life (25–27), limited life expectancy (28), and premature death (29, 30). Although research into frailty is continuously developing, some pillars of the condition have been established: it is an age-related condition (25, 31), though it is not an inevitable consequence of the process of aging (3, 32, 33). It is multidimensional, that is, it affects multiple aspects of health, namely physical, psychological, cognitive, social, emotional, spiritual, economic (32, 34–36), and nutritional domains (37). It is a dynamic and reversible state, at least in its initial stages (38), that is, persons can fluctuate from a robust state to a state of frailty, until the reduction in their physiological reserve prevents recovery to their baseline status (39). In addition, transitioning to more severe levels of frailty is more common than reversing frailty (3). As frailty mirrors biological rather than chronological age (40, 41), identifying biomarkers of this condition is important. However, to date, biomarkers that reflect biological age better than chronological age are as yet unavailable (42). These could also help identify frailty objectively and could contribute to further understanding its pathophysiology.

## The Epidemiology of Frailty

The prevalence of frailty is higher (up to two-fold) in women, compared to men (18). Among community-dwelling older adults (≥65 years), the prevalence of frailty ranges between 5 and 16% (40, 43, 44), depending on the definition used and population examined; this percentage is known to be higher in clinical settings (38), reaching 85% in nursing home residents (40, 45, 46). Age is strongly associated with frailty; the American Medical Association reported that 40% of people aged ≥80-year-old are frail (47). Similarly, centenarian studies report rates over 50% (48). Given the extent of population aging, the association between frailty and age and the impact of frailty on adverse outcomes for older people, it is increasingly being recognized to be a significant public health concern (6, 7).

## Frailty Models

The two most widely-used approaches to classify frailty are the physical frailty and deficit accumulation models of frailty. The Frailty Physical Phenotype was initially proposed by Fried et al. (18) who used the Cardiovascular Health Study to define the “frailty phenotype,” identifying five physical components, specifically exhaustion (self-reported), low physical activity, weakness (low grip strength), slow gait speed, and shrinking (unintentional weight loss ≥5% in prior year), whose presence in number ≥3 establish physical frailty. If only 1 or 2 criteria are met, persons are defined prefrail, while when no components are present, persons are deemed robust. Rockwood and Mitnitski (49) and Mitnitski et al. (50) validated the cumulative deficit model using data from the Canadian Study of Health and Aging to create a Frailty Index, which originally included a total of 70 deficits including signs, symptoms, disabilities, diseases, and laboratory investigations (21). The cumulative number of disorders present in a person is divided by the total items explored: the more deficits are present, the higher the possibility that the individual is frail (51). Different items, consisting of activities of daily living, diseases, impairments, can be present in the frailty index, since not all of the deficits need to be necessarily considered, and a subset can be used (52). While these two operational models are the most commonly applied frailty constructs, they are different and should be considered complementary, rather than alternatives (52). It is worth pointing out that neither capture all aspects of frailty; the frailty phenotype almost exclusively assesses physical frailty, while the frailty index captures multi-morbidity but does not clearly distinguish frailty from disability (52). Frailty is more complex than the presence of multiple deficits, limitations in activities of daily living or physical deficits alone. It also incorporates elements relating to the functional reserve, psychology and social environment of the individual. More recently, a broad range of screening and assessment instruments such as the Comprehensive Frailty Assessment Instrument (53) and the Tilburg Frailty Index (54) have been developed taking a more biopsychosocial approach (55). This characterizes frailty as a dynamic state resulting from deficits in any of the physical, psychological and social domains, which contribute to health. This conceptual model along with the physical and deficit accumulation models of frailty also lacks a specific operational definition.

## Frailty and Sarcopenia

Many of the pathophysiological changes resulting in frailty remain unclear (32). In the late 1980s, Rosenberg invented the term sarcopenia to describe the loss of lean body mass experienced with aging (56). One of the first theories linking frailty and sarcopenia dates back to 1994, when Fiatarone et al. hypothesized a connection between frailty and decline in muscle mass, suggesting that improvement in muscle mass may be beneficial for people experiencing frailty (57). While an association between sarcopenia and frailty has been established, the pathophysiology of frailty appears to be more complex than the effect of sarcopenia alone (58–60). For example, it is still not clear whether sarcopenia causes frailty or whether it is a manifestation of it. Like frailty, sarcopenia is more prevalent among older adults, is associated with adverse outcomes and is potentially reversible (61). Both conditions can lead to functional decline and disability; thus, the early detection of both is recommended (61). To further study this connection, the ongoing study by Calvani et al. “BIOmarkers associated with Sarcopenia and PHysical frailty in EldeRly pErsons” (BIOSPHERE) proposes to identify biological markers for sarcopenia and physical frailty through blood sample analysis and may help shed some light on the connections between frailty and sarcopenia (6, 62). A better understanding of the two conditions and the connection between them could help in their prevention and management.

## Frailty and Multimorbidity

The epidemiological transition has led to prolonged life expectancy. This has resulted in chronic rather than acute conditions becoming the leading cause of morbidity and mortality (63). As a result, older adults are increasingly facing multimorbidity and chronic illnesses, such as diabetes, hypertension, and dementia as they age (64). Multimorbidity is defined as the presence of two or more chronic diseases (65, 66). Its impact on the health status is dependent on the interaction between the specific illnesses that affect the individual simultaneously (67), which is higher than the sum of the single effects expected from each disease (68). Frailty and multimorbidity are two overlapping but distinct conditions (19, 58, 69) and therefore require different management (69, 70) and prevention strategies. Although, the presence of multiple chronic conditions is associated with the development of frailty (6, 18), it is not necessarily the consequence of chronic disease. Frailty can be present even in the absence of chronic conditions, suggesting that there are different pathways leading to its development (71). Evidence showing how multimorbidity can lead to frailty is still lacking (72, 73) and more research on this is needed. The prevalence of both conditions increases with age, even if they do not affect only older persons (40, 74). However, multimorbidity is more ubiquitous than frailty, with up to three out of four people aged ≥75 years, fulfilling the criteria for multi-morbid (75–77). Furthermore, chronic diseases and frailty predict disability (16), and are associated with adverse outcomes and worse prognosis when they are both present (78).

As identifying frailty is considered a more reliable predictor of adverse outcomes than multimorbidity (79), the National Institute for Health and Care Excellence and the British Geriatric Society underline the importance of recognizing frailty in older adults with multimorbidity as these are at greater risk of adverse outcomes (80, 81). This could help target and rationalize appropriate treatment of multimorbidity, given that evidence suggests that intensive or over-treatment of chronic diseases may increase negative health outcomes in frail people (67, 82). Furthermore, when caring for persons with frailty and multimorbidity, it is important to take into account that frailty could hinder adherence to both pharmacological and physical therapies (83).

## Frailty and Cognitive Impairment

Within the biopsychosocial model of frailty, research exploring the cognitive and psychological aspects of the syndrome have highlighted the relationship between physical frailty and cognitive impairment, leading to the conceptualization and operationalization of cognitive frailty (84). Experts from the International Academy of Nutrition and Aging found consensus on the definition of cognitive frailty, identifying it as a state that requires the presence of physical pre-frailty or frailty [according to the Frailty Phenotype (18)], and mild cognitive impairment (MCI), diagnosed with questionable dementia on the Clinical Dementia Rating (CDR) (score 0.5), a state similar to MCI (85). More recently, using these criteria, two main subtypes of cognitive frailty have been proposed: reversible cognitive frailty representing subjective cognitive decline (i.e., pre-MCI, a CDR score = 0) and potentially reversible cognitive frailty, equivalent to MCI (a CDR score = 0.5) (86–88). Both subtypes require the co-occurrence of physical pre/frailty and studies have shown that gait speed or grip strength are the physical characteristics most frequently associated with cognitive frailty (89). Different instruments other than the CDR have been used to detect cognitive impairment (90), leading to different operational definitions of cognitive frailty (88). Hence as with general frailty, more research is needed to develop a shared operational definition. As for physical frailty, the onset of cognitive frailty can be delayed and reversed, at least in the first stages, and the condition can lead to a higher risk of adverse health outcomes (26), namely disability (91), worsened quality of life (92), hospitalization and mortality (93). Thus, public health professionals favor a life-course approach, intervening at a younger age with preventative strategies including physical activity and dietary modification, e.g., the Mediterranean diet (88). These may help prevent or delay onset of not only cognitive frailty but also sarcopenia and physical frailty (94), though further research is needed to confirm such findings.

## Frailty and the Social Determinants of Health

In a broader sense, health reflects multiple factors beyond the medical, including social, economic, political, and environmental; individuals are affected by multiple environmental and social factors, which impact on their health (95), and contribute to social vulnerability (96). Furthermore, the social determinants of health, namely education, housing, job, nutrition, environmental protection (97), can decrease an individual's intrinsic and extrinsic capacity, rendering them frail. The ability of the individual and their social and caregiver network to manage frailty influences the risk of a broad range of adverse outcomes, including institutionalization and death, more so than their medical conditions or their ability to perform daily activities (98). Thus, as the manifestations of frailty are evident across not only the physical and the psychological, but also the social domain, it is important to explore the social consequences of frailty. Although patient-centered care is increasingly at the core of managing health, more research into the relationship between frailty and social vulnerability is needed so that an individual's social environment, perspective and desires are not overlooked. The work by Azzopardi et al. highlights how even within the biopsychosocial approach toward frailty, the social aspects and especially the environmental and personal factors (e.g., relationships) of the individual are not adequately considered by health and social care professionals (96). A recent scoping review examining the construct of social frailty (i.e., inadequate social resources to fulfill an individuals' social needs) found that the loss of social activities and self-management abilities are important components and must be supported to minimize adverse outcomes (99). Because public health professionals have a broader view of health determinants compared to clinicians, they are in a privileged position to address the social needs of people and to intervene in their home environment.

## Screening and Assessing for Frailty

At least in its initial stages, before the onset of functional impairment, frailty is often reversible (5, 38). Hence, early identification, usually at the pre-frail stage (100), is important to help individuals regain function and to prevent negative outcomes associated with the syndrome. Despite the importance of diagnosing frailty, there is no definitive evidence or consensus as to whether screening should be routinely implemented in different settings, whether a certain age threshold should be set (58, 101), nor which domains should be investigated (36, 102). Indeed, there is little evidence to support primary healthcare services for population-level screening, surveillance or monitoring of frailty (103). Nonetheless, the Royal College of Physicians, the French Society of Geriatrics and Gerontology (104, 105) and the British Geriatrics Society (80) all recommend opportunistic or targeted screening for frailty. Several short instruments to screen and assess frailty exist, although a standard approach to define frailty has yet to be agreed, hampering the ability to measure it (5, 31, 106). Given the multidimensional characteristics of the syndrome, various instruments with different features are validated and can be used depending on the clinical setting. Among these instruments, some are intended only to detect physical frailty while others are more multidimensional (Table 1). The main limitation of all these instruments is that they do not suggest any intervention on the basis of their score.

**Table 1:** Selection of frailty screening and assessment instruments comparing uni and multidimensional scales.

**Unidimensional**	**Multi-domain and multidimensional**
Frailty Phenotype	Frailty Index
Gait Speed	Clinical Frailty Scale
Timed-Up-and-Go Test	Groningen Frailty Indicator
INTER-FRAIL	Edmonton Frail Scale
Short Physical Performance Battery	Gérontopôle Frailty Screening Tool
FRAIL scale	Frail Elderly Functional Assessment
	PRISMA-7 Questionnaire
	Kihon Checklist
	Tilburg Frailty Index
	Comprehensive Frailty Assessment Instrument

*The “Frailty Phenotype” (18), the “Gait Speed” (131), the “Timed-Up-and-Go Test” (132), the “INTER-FRAIL” (133), the “Short Physical Performance Battery” (134), the “Frail Scale” (58) the “Frailty Index” (49, 50), the “Clinical Frailty Scale” (21), the “Groningen Frailty Indicator” (135), the “Edmonton Frail Scale” (136), the “Gérontopôle Frailty Screening Tool” (137), the “Frail Elderly Functional Assessment” (138), the “PRISMA-7 Questionnaire” (139), the “Kihon Checklist” (140), the “Tilburg Frailty Index” (54), and the Comprehensive Frailty Assessment Instrument (53)*.

To date, Comprehensive Geriatric Assessment (CGA) is considered to be the gold standard to assess frailty (3). However, it was not designed for this purpose and may not necessarily completely represent frailty, as it was originally intended to detect disability (69, 70). For this reason, a modified CGA might be more meaningful to use to identify frailty (80, 107). CGA is nevertheless important to create tailored interventions, though it can be time consuming and requires specialist input. PHC may be the best place to screen for frailty (80, 108, 109). To this end, it is worth stressing that PHC physicians and specialists should receive appropriate training on how to detect frailty (69), in order to appropriately screen for the condition (58). There are an increasing number of examples of frailty education programs for healthcare professionals (110). These are supported by guidelines on interprofessional learning, which is central to frailty education (111). For example, in Ireland, the National Frailty Education Programme, which aims to provide education on the key fundamentals of frailty to a wide range of healthcare professionals in all healthcare settings, was successfully implemented (112). There is a need for a similar approach in other countries to provide the necessary skills to healthcare professionals and enhance their understanding of frailty and ensure early identification and appropriate management of this condition.

## Frailty and COVID-19

In light of the pandemic caused by severe acute respiratory syndrome coronavirus 2 (SARS-CoV-2), prognostic factors to allocate resources in case of limited medical supplies have been at the center of the discussion worldwide. Studies have shown that increasing age is associated with adverse outcomes for individuals with COVID-19 (113, 114). Given this, some have favored the use of age as a prognostic factor. However, recent evidence (115) suggests that age alone is insufficient, and that frailty may help guide in the evaluation of those most likely to benefit from critical care (116). For this reason, the National Institute for Health and Care Excellence (NICE) (117) recommends assessing frailty in all older adults with COVID-19 (except those with stable long-term disability such as cerebral palsy). The Clinical Frailty Scale (CFS) can be used with caution to support clinical decision-making for older adults with COVID-19 (116). The COVID-19 in Older PEople (COPE) study evaluated the utility of the CFS and showed that frailty predicts earlier death and longer time spent in hospital (118). This new evidence points to the central role that frailty assumes when caring for older persons with acute illness. Despite this, only limited data are available to support the use of frailty or any specific screening instrument at population-level in order to predict the severity of the clinical manifestations of COVID-19 in older people. As the pandemic continues, more data are required to help researchers, clinicians and public health officials to better understand how identifying frailty among older adults at a primary care level could help mitigate the most negative consequences of the pandemic for older populations.

## Discussion

This paper examines some of the key public health aspects of frailty. The need to understand the concept from a public health perspective is being increasingly recognized, such that frailty has been labeled as the “future core business of public health” (119) While several definitions of frailty are available these as yet, do not help operationalize the concept (5). This has limited the uptake of frailty as a research construct among public health professionals who have up to now focused more on risk factors for adverse aging including non-communicable chronic disease and socioeconomic inequalities. Involving international experts from different settings amongst health and social care professionals, academics and older people themselves could be the first step to reach a general agreement on a definition. Furthermore, consensus on the dimensions that must be examined for an operational definition has not been reached yet (102). Given the heterogeneity between definitions used to classify frailty and the varied features of frailty, the same person could be both frail and not frail, due to the different domains investigated. We suggest that reaching consensus on an operational definition might not necessarily mean finding a unique definition that fits for all health and social care settings, but rather having a common understanding and a multidimensional approach on ways to define and recognize the condition among all professionals including public health professionals is required. Contextual terms such as social frailty, nutritional frailty, physical frailty, and cognitive frailty may also be useful to improve uptake of the concept of vulnerability to adverse events that is at the core of frailty (34, 84).

At present, there is little data available for screening and assessing frailty at population-level (103). Furthermore, we suggest that implementing population-level screening would require upskilling the existing workforce and further research evaluating both the effectiveness and cost-effectiveness of this approach at a primary care and public health level. The role of secondary care (e.g., hospital-based Geriatricians) and how this could support community-based services also needs to be clarified. Identifying and labeling individuals as frail without clear benefit risks itself causing harm. Defining a concept influences how we identify it (34), and gives it a clear significance, with repercussions in everyday life. Labeling persons as frail might have consequences on how society relates to and engages with them (120). This could affect how people perceive themselves and their role in society as well as in the familial context. It is important that, even when recognized as frail, people are able to feel valuable and participate in everyday life to the fullest of their capabilities. To achieve this goal, as a society, we need to create environments that allow frail people to feel socially involved, minimizing social stigma. Hence, frailty does not only concern health-care services, but also social services and communities in their entirety as well. There is also a need to provide better communication to individuals with frailty and their supports in order to help people to contribute irrespective of their level of frailty in every aspect of life in so much as is possible. This an important approach to frailty that PHC and public health professionals should strive to achieve. Communication with the public regarding frailty is also needed. Public health campaigns explaining that this is a condition influenced by the life course is important. Therefore, as it is important to identify frailty at an early stage, we assert that more frailty research including people younger than 65 is required, as this would help identify frailty in its earliest, prodromal stage, usually referred to as pre-frailty (106, 121). Public health campaigns combined with interventions targeting pre-frail individuals may provide more positive outcomes, given that the reserve capacities at this stage are still sufficient to preserve functional abilities (122).

Population-level interventions taking a public health approach, centered on education are also appropriate. Again these should begin early and target younger individuals before the onset of frailty.

Although it is not yet clear how best to target frailty, the biopsychosocial model is the most appropriate to provide a holistic assessment of the patient. Recognizing which domains (i.e., physical, cognitive, nutritional, psychological, social, economic) contribute to the loss of function would serve as a proxy for health-care utilization and improve the quality of patient-centered care (123), favoring targeted strategies for prevention and management at population-level. Education is vital to ensuring that providers and older people themselves are well-placed to take advantage of these approaches. Evidence suggests knowledge about the prevention and reversibility, “malleability,” of frailty is poor (123); hence, in the context of aging societies worldwide and high levels of frailty in all countries (124), there is a need to increase awareness at all levels (i.e., micro, meso, macro). In this sense, frailty should no longer be confined to geriatric medicine settings. For example, most healthcare specialties manage older people with complex needs that require a broader understanding of the overall health status of the patient (68), rather than a disease-specific approach. Furthermore, even if impairment is detected in a specific domain, the increased vulnerability associated with frailty places individuals at higher risk for rapid deterioration in other domains too. This requires proactivity rather than reactivity to prevent this from happening and the adoption of a person-centered, community-focused public health model. For this purpose, a holistic approach is needed when caring for frail older people. Thus, public health personnel should be first introduced to the multidimensional nature of frailty, then trained to identify frailty and consequently made aware of that it is not merely a clinical concept, but also a societal issue that can be addressed in the individuals' environment and within their social relationships. Moreover, public health professionals can contribute to education and training on frailty at a community level, fostering community-based interventions to support older adults and their caregivers to prevent and manage frailty. Similarly, policy-makers need to be more conscious of the role frailty plays and to shape policies that help foster seamless care for those with complex needs and to increase the ability of people to self-manage (125). The need to provide integrated care at population-health level is particularly important (126). Fragmentation of care hinders the possibility of properly addressing each aspect of frail individuals' complex needs.

The influence of socioeconomic inequalities on the development of frailty and outcomes amongst frail individuals cannot be underestimated. Frailty is usually associated with a lower socioeconomic status; frail individuals are usually less educated and have lower incomes (18, 40). This underlines how health is highly influenced by social factors. Furthermore, the lack of shared assessment of environmental and social factors, that are sparingly reported in currently multidomain frailty instruments, could influence a misleading approach to fulfill the real needs of frail individuals and populations (96). Services need to be able to intervene to address the social determinants of health, which too often, particularly in healthcare settings, are not considered as an integral part of the well-being of the person. Traditional health-care systems with their siloed structure and a strong hospital-centric and cure-first culture, need to be re-focused to adapt to the new complex and chronic care needs of populations. For this purpose, we need to put into action the framework for the redesign of healthcare centered around PHC set out in 1978 in the Alma-Ata Declaration and reaffirmed in 2018 in the Astana declaration (127). Public health, PHC and social services must be at the center of managing the care of frail older adults, promoting integrated care and a life-course approach to health. Intermediate care, which has been developed to foster the integration between acute and PHC and provides a wide range of both health and social services to bridge care for older and frail persons who have complex needs (128), could foster the management of complex needs of frail adults. It has been shown to influence healthcare outcomes including hospitalizations, though further study particularly at population-level is required (129). Hence, while it has been said that “complex problems require complex solutions” (130), we assert that complex needs require holistic and integrated care.

## Conclusions

Further research is needed to address the challenging task of finding consensus on the conceptual and operational definition of frailty. This is important to support its use in public health. While physical frailty has been seen as the core feature of the condition, frailty is a more complex, and multidimensional syndrome influenced by the social determinants of health. Population aging is a worldwide trend that is no longer limited to more economically developed countries. Therefore, thorough study on the possible causes, role of socioeconomic factors, the high prevalence in low- and middle-income countries, and on possible prevention and management strategies that can be adopted in countries with less resources compared to developed countries, should be fostered. The orientation of the PHC vision outlined in the Alma-Ata declaration lays the foundation of what is known today as integrated care. Forty-two years later, it is time for public health and PHC to become the fulcrum of care through health promotion and prevention activities in the community, and through new models of care that foster the integration of health and social care professionals, guaranteeing a holistic person-centered approach to the complex needs of frail people.

## Author Contributions

The authors conceived and shared the idea of this manuscript and equally contributed to its content.

## Conflict of Interest

The authors declare that the research was conducted in the absence of any commercial or financial relationships that could be construed as a potential conflict of interest.
